# Transcriptomic and functional analyses on a *Botrytis cinerea* multidrug‐resistant (MDR) strain provides new insights into the potential molecular mechanisms of MDR and fitness

**DOI:** 10.1111/mpp.70004

**Published:** 2024-09-07

**Authors:** Georgios Sofianos, Edoardo Piombo, Mukesh Dubey, Magnus Karlsson, George Karaoglanidis, Georgios Tzelepis

**Affiliations:** ^1^ Faculty of Agriculture, Forestry and Natural Environment, Laboratory of Plant Pathology Aristotelian University of Thessaloniki Thessaloniki Greece; ^2^ Department of Forest Mycology and Plant Pathology Swedish University of Agricultural Sciences, Uppsala Biocenter Uppsala Sweden

**Keywords:** *Botrytis cinerea*, fitness, MFS‐transporters, multidrug resistance, virulence

## Abstract

*Botrytis cinerea* is a notorious pathogen causing pre‐ and post‐harvest spoilage in many economically important crops. Excessive application of site‐specific fungicides to control the pathogen has led to the selection of strains possessing target site alterations associated with resistance to these fungicides and/or strains overexpressing efflux transporters associated with multidrug resistance (MDR). MDR in *B. cinerea* has been correlated with the overexpression of *atrB* and *mfsM2*, encoding an ATP‐binding cassette (ABC) and a major facilitator superfamily (MFS) transporter, respectively. However, it remains unknown whether other transporters may also contribute to the MDR phenotype. In the current study, the transcriptome of a *B*. *cinerea* multidrug‐resistant (MDR) field strain was analysed upon exposure to the fungicide fludioxonil, and compared to the B05.10 reference strain. The transcriptome of this field strain displayed significant differences as compared to B05.10, including genes involved in sugar membrane transport, toxin production and virulence. Among the induced genes in the field strain, even before exposure to fludioxonil, were several putatively encoding ABC and MFS transmembrane transporters. Overexpression of a highly induced MFS transporter gene in the B05.10 strain led to an increased tolerance to the fungicides fluopyram and boscalid, indicating an involvement in efflux transport of these compounds. Overall, the data from this study give insights towards better understanding the molecular mechanisms involved in MDR and fitness cost, contributing to the development of more efficient control strategies against this pathogen.

## Graphical abstract

A *Botrytis cinerea* multidrug‐resistant field strain shows induction of major transmembrane transporter genes and involvement of an major facilitator superfamily transporter in tolerance to certain fungicides.

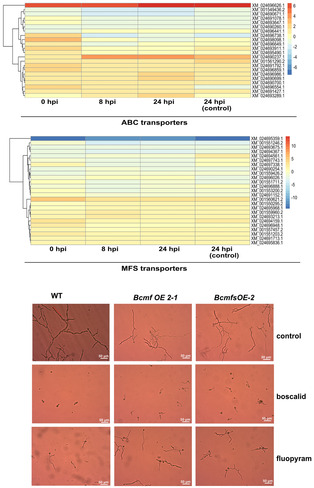

## 1 INTRODUCTION

*Botrytis cinerea* is one of the most destructive pathogens of greenhouse‐grown vegetables, grapes and small berry fruits, causing pre‐ or post‐harvest rots (Droby & Lichter, 2007). In addition, it can cause severe post‐harvest diseases in fruits such as apple, pear, kiwifruit and ornamental plants such as roses, with severe yield losses worldwide (Droby & Lichter, 2007). In rootstock nurseries, *B. cinerea* is a major problem as well, infecting all stages of seedling growth, seeds and plantlets (Mittal et al., 1987). Inoculum is usually conidia, mycelia or sclerotia that can be disseminated by wind, irrigation or rainwater, human activities, and can be easily spread in the whole greenhouse, nursery or orchard (Kerssies et al., 1997). It enters the plant tissues mostly at the earlier developmental stages and stays quiescent for a considerable period, while symptoms become mostly evident either before harvest or in storage facilities after harvest (Bi et al., 2023; Hua et al., 2018).

Because *B. cinerea* employs different modes of action to infect its broad host range and forms sclerotia, to survive under harsh environmental conditions over a long period of time, the management of this pathogen is challenging (Williamson et al., 2007). Control of grey mould disease is based mostly on chemical treatments, and several fungicides with different modes of action have been registered so far (Hahn, 2014). Among them, fungicides inhibiting cell respiration, such as succinate dehydrogenase inhibitors (SDHIs), (i.e., boscalid and fluopyram) or quinone outside inhibitors (QoIs), (i.e., pyraclostrobin and azoxystrobin); fungicides with other mitochondrial modes of action, such as anilinopyrimidines (i.e., cyprodinil and pyrimethanil) (Mosbach et al., 2017); fungicides targeting fungal osmoregulatory signal transmission pathways, such as phenylpyrroles (i.e., fludioxonil), and fungicides inhibiting sterol biosynthesis (i.e., fenhexamid), have commonly been used against *B. cinerea* (Hahn, 2014).

However, the extensive and repeated application of fungicides in combination with some *B. cinerea* biological traits such as the short life cycle, the high genetic variability and the high rates of asexual reproduction, increase the risk for fungicide resistance development (Leroux et al., 2002). In fact, *B. cinerea* strains resistant against a variety of fungicides have been reported, dramatically reducing fungicide efficacy (De Miccolis Angelini et al., 2014; Harper et al., 2022; Konstantinou et al., 2015; Malandrakis et al., 2022; Naegele et al., 2022; Notsu et al., 2021; Samaras et al., 2016; Veloukas et al., 2014). The most common fungicide resistance mechanism in plant pathogens is the alteration of the target site due to point mutations in the corresponding target genes. These mutations lead to a change in the corresponding protein's binding site, thus reducing its affinity to the active ingredients. Worldwide, there have been observed *B. cinerea* populations that have developed resistance against every site‐specific fungicide (Fernandez‐Ortuno et al., 2017; Hahn, 2014; Kim & Xiao, 2011; Saito et al., 2014; Sang et al., 2018). While the mutated proteins may remain operative for the cell, it is common that some mutations may hamper the proteins' functionality and lead to unfavourable phenotypic changes, known as fitness cost or fitness penalty in the absence of fungicide pressure (De Miccolis Angelini et al., 2015). In *B. cinerea* field populations, several resistance‐conferring mutations have been correlated with fitness defects. While there exist certain mutations that do not seem to affect the strains' fitness, most mutations can lead to reduced virulence, conidiation, mycelial growth and sclerotia survival (Billard et al., 2011; Veloukas et al., 2014).

Apart from target site alterations, there are also other mechanisms that can give rise to fungicide resistance. The most common non‐specific resistance is referred to multidrug resistance (MDR), caused by the overexpression of ATP‐binding cassette (ABC) and major facilitator superfamily (MFS) transporters that transport molecules across the plasma membrane, and efflux them outside the cytoplasm (Coleman & Mylonakis, 2009; Dos Santos et al., 2014). ABC transporters are grouped in distinct phylogenetic groups (A–H), and those belonging to groups B, C and G are referred to as multidrug resistance (MDR), multidrug resistance‐associated proteins (MRP) and pleiotropic drug resistance (PDR) transporters (Kovalchuk & Driessen, 2010; Paumi et al., 2009). Regarding the MFS transporters, they form the largest superfamily being characterized until now, with over 100 families (Wang et al., 2020).

The role of ABC/MFS transporters in efflux of a variety of chemical compounds in fungal cells is well known (Hayashi et al., 2002a, 2002b; Rafiei et al., 2022; Samaras et al., 2020; Stefanato et al., 2009), and fungal genomes of species with increased tolerance to xenobiotic substances show expansion of membrane transporter gene families (Karlsson et al., 2015; Nygren et al., 2018). Concerning *B. cinerea*, some MDR types have already been characterized. More precisely, four different MDR phenotypes exist, namely MDR1, MDR2, MDR3 and a subclass of the MDR1 phenotype with enhanced resistance levels namely MDR1h (Kretschmer et al., 2009; Leroch et al., 2013). *B. cinerea* strains expressing the MDR1 phenotype seem to constitutively overexpress the ABC transporter *BcatrB* and are partially tolerant to fludioxonil and cyprodinil (Kretschmer et al., 2009). In MDR1h strains, the expression levels of *BcatrB* are generally higher compared to that of MDR1 and this leads to higher levels of resistance to fungicides (Fernandez‐Ortuño et al., 2015; Leroch et al., 2013). MDR2 strains constitutively overexpress the MFS transporter *mfsM2* and are partially resistant to fenhexamid, cyprodinil and iprodione (Kretschmer et al., 2009). The MDR3 phenotype is a combination of MDR1 and MDR2 phenotypes with widened spectrum and resistance levels (Kretschmer et al., 2009; Leroch et al., 2013).

In a recent study, aiming to determine MDR types and their respective frequencies in *B. cinerea* populations originating from crops heavily treated with fungicides in Greece, it was found that MDR1h was the predominant MDR type (Sofianos et al., 2023). MDR1h strains are characterized by an increased activity of *BcatrB* associated with a 3‐bp deletion at codon 497 in the *mrr1* exon and several peptide sequence alterations in the produced protein. However, it remains unknown whether other ABC or MFS transporters in field strains, selected through time in the heavily treated crops, contribute with their increased expression to the observed fungicide resistance phenotype. Thus, in the current study we hypothesized that such strains could overexpress numerous uncharacterized ABC and/or MFS transporters that could potentially increase their tolerance to fungicides. To test that, a transcriptomic analysis between the *B. cinerea* MDR1h field strain Ap2 and the non‐MDR B05.10 reference strain was conducted. It revealed that these two strains displayed significant transcriptomic differences, especially in genes related to glucose transport, virulence and toxin biosynthesis. Follow‐up experiments showed that this MDR strain grew better in carbon‐minimal media, while reduced virulence was also observed. In addition to *BcatrB*, several other ABC and MFS transporters were constitutively induced. Among them, a previously uncharacterized MFS one was selected for further functional characterization; we identified that it possibly contributes to tolerance to SDHIs. Overall, this study gives new insights towards better understanding of MDR in filamentous fungi and the potential mechanisms associated with the fitness of fungal strains.

## 2 RESULTS

### 2.1 The transcriptomes of Ap2 and B05.10 significantly differ

**FIGURE 1:**
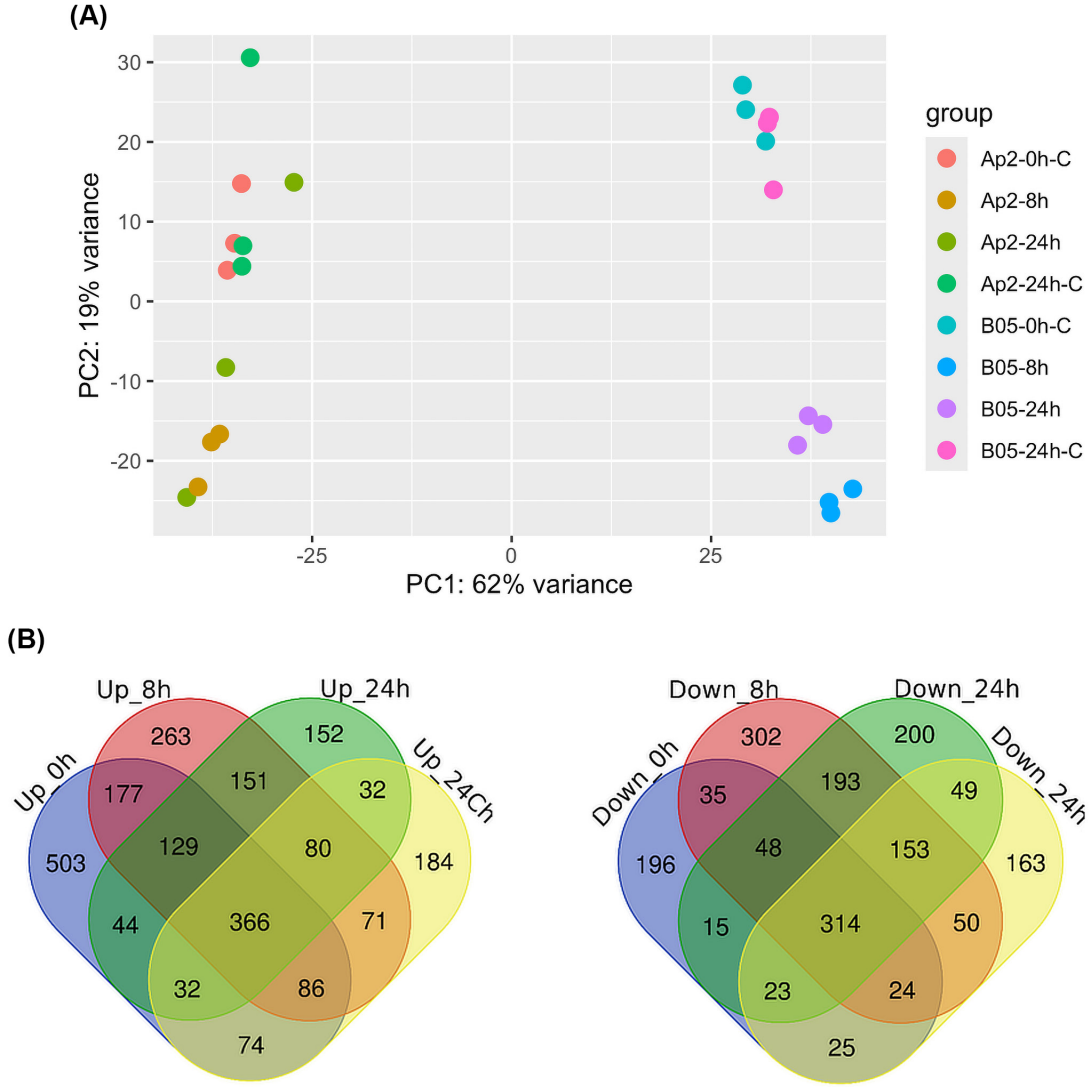
The transcriptomes of Ap2 and B05.10 significantly differ. (a) Principal component analysis plot of gene expression data after variance‐stabilizing transformation, showing the relationship between the Ap2 and B05.10 strains upon exposure to fludioxonil. (b) Venn diagrams showing the number of differentially expressed genes between the Ap2 and B05.10 strains upon exposure to the fungicide fludioxonil. The 0 and 24 h post‐inoculation (hpi)‐C samples represent no fungicide exposure and used as controls.

Τo investigate the genetic basis of the MDR phenotype, a comparative transcriptomic analysis was conducted between the Ap2 and B05.10 strains during exposure to fludioxonil. An exploratory principal component analysis (PCA) showed that the difference between the Ap2 and B05.10 strains accounted for most of the variance in the dataset, with PC1 (62% of the variance) clearly separating samples according to genotype. Nevertheless, PC2 seemed to account for the effect of fludioxonil, with most treated samples clustering separately from the control ones, especially in the case of the B05.10 strain (Figure 1a).

The differential gene expression analysis showed how 503 genes were uniquely up‐regulated and 196 were down‐regulated at 0 hpi (no exposure) between these two strains (Figure 1b). Interestingly, the number of uniquely up‐regulated genes was significantly reduced at 24 hpi (152 genes), while the number of down‐regulated genes remained almost unchanged (Figure 1b). After 8 h of exposure to fludioxonil, 263 genes were up‐regulated, while 302 genes were down‐regulated (Figure 1b). After exposure for 24 h, the number of up‐regulated genes was reduced to 152, while 200 genes were down‐regulated (Figure 1b). The gene ontology (GO) analysis on all differentially expressed genes (DEGs) showed, among others, up‐regulation of genes in Ap2 involved in “glycerol and glucose import and transmembrane transport” (for instance, GO: 0015793, GO:1904659, and GO:0046323) (Figure S1).

**FIGURE 2:**
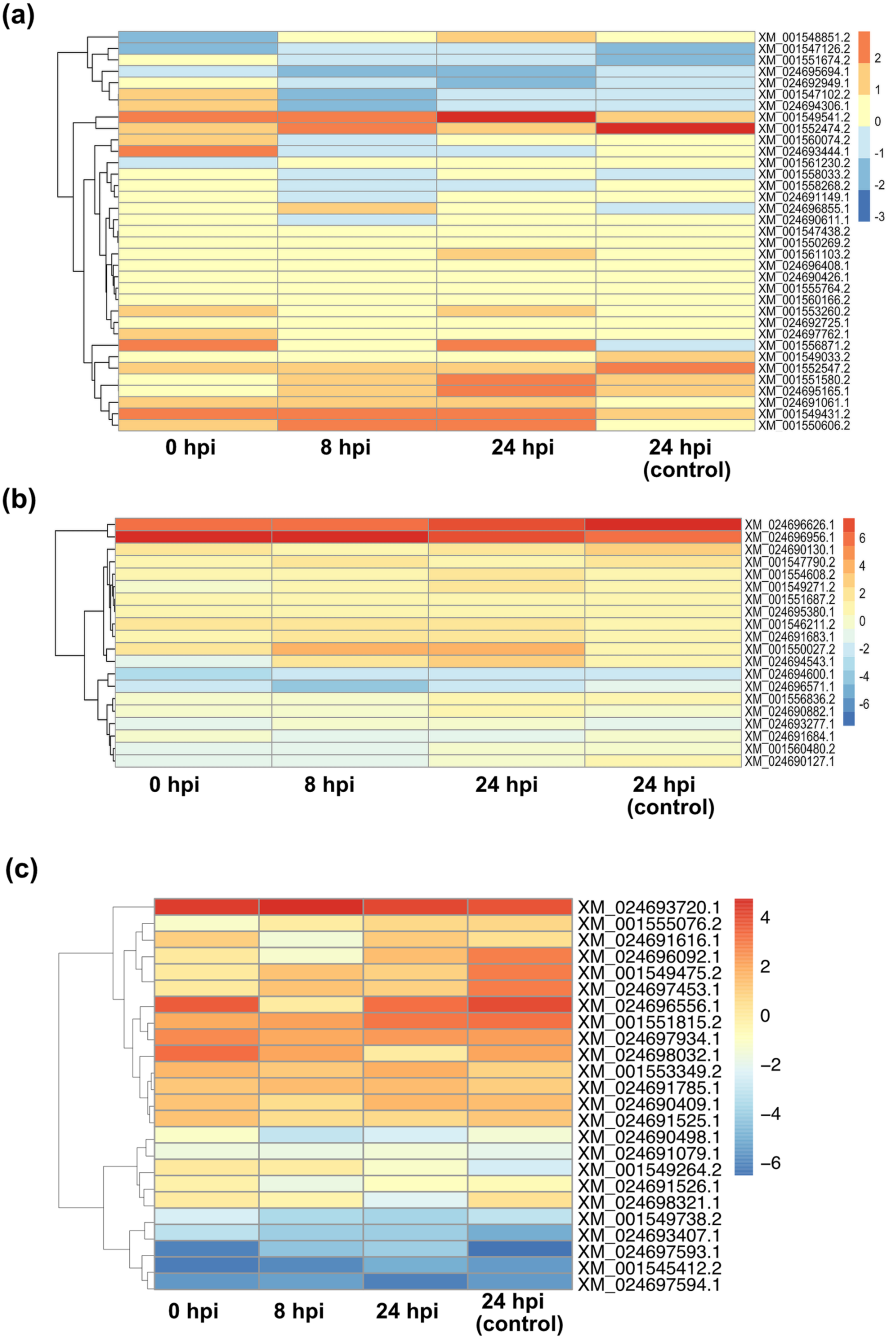
The *Botrytis cinerea* multidrug‐resistant Ap2 strain shows differentially expressed genes involved in (a) glucose transport, (b) virulence and (c) toxin biosynthesis, as compared to B05.10. Analysis was conducted upon fungal exposure to the fungicide fludioxonil and the 0 and 24 h post‐inoculation (hpi)‐(control) samples represent no fungicide exposure and used as controls. (Adjusted *p*‐value <0.05, absolute log_2_ fold‐change >2 for up‐regulated genes and <−2 for the down‐regulated ones). Red and blue colours represent up‐regulated and down‐regulated genes, respectively.

Following the GO analysis, we focused on particular DEGs in each category. We found that 35 genes putatively encoding glucose transporters were differentially expressed, with four genes most highly induced (XM_001549541.2, XM_024693444.1, XM_001556871.2, XM_001549431.2) and four genes down‐regulated (XM_001548851.1, XM_001547126.2, XM_024695694.1, XM_001561230.2) in the Ap2 strain as compared to B05.10 before fungicide exposure (Figure 2a; Table S1). Among them, eight genes were induced at all tested time points (Figure 2a; Table S1). In order to investigate whether induction of these glucose transporter genes has any impact on mycelial growth, the *B. cinerea* strains were inoculated in media with different carbon concentrations: rich (V8), intermediate (IM) and minimal (MM). Our results showed that Ap2 displayed a faster growth in all tested media as compared to B05.10 (Figure S2a). Interestingly, we observed that the strongest effect on mycelial growth rate was observed on MM, where carbon availability was limited (*p* < 0.001) (Figure S2a), showing a possible correlation between induction of these genes and fungal growth. Finally, down‐regulation of genes involved in “DNA repair” mechanisms (for instance, GO: 0000724 and GO: 0006302), as compared to the B05.10 strain, was observed (Figure S1).

### 2.2 Certain genes involved in *B. cinerea* virulence are suppressed in the MDR strain

Further analysis on genes encoding for virulence factors identified 20 genes that were differentially regulated in Ap2 as compared to B05.10 in all the tested conditions (Figure 2b; Table S1). Among them, genes with a confirmed involvement in *B. cinerea* virulence were down‐regulated in the Ap2 strain, such as the *Bcbot1* gene (*XM_024696571.1*), encoding a P450 monooxygenase involved in the botrydial pathway (Pinedo et al., 2008; Siewers et al., 2005) (Figure 2b; Table S1). Furthermore, the gene *Bcboa2* (*XM_024690127.1*), encoding a polyketide synthase (PKS) with a crucial role in the synthesis of the phytotoxin botcinic acid (Dalmais et al., 2011), was also suppressed in the Ap2 strain (Figure 2b; Table S1). In addition, down‐regulation of the *Bcaba2* gene (*XM_024694600.1*), encoding a protein with monooxygenase activity, being involved in abscisic acid synthesis and fungal virulence (Siewers et al., 2005; Zhang et al., 2016), was also observed in this strain (Figure 2b; Table S1). In contrast, two genes with confirmed roles in fungal virulence were induced in Ap2 in all tested conditions; one of them was the *Bcpg2* gene (*XM_024696956.1*), encoding a polygalacturonase enzyme, and the other was *BcatrB*, encoding an ABC transporter (*XM_024696626.1*) (Kars et al., 2005; Stefanato et al., 2009).

To investigate the virulence of the Ap2 and B05.10 strains, *Arabidopsis thaliana* plants were infected and the symptom development was evaluated. Our results showed that leaves infected with conidia derived from the Ap2 strain displayed smaller lesions and less severe symptoms (*p* < 0.001) as compared to plants infected with the B05.10 strain 3 days post‐inoculation (dpi) (Figure S2b,c). The observed lower virulence of Ap2 strain maybe correlated with the reduced transcription of the above genes. Finally, our transcriptomic analysis showed that 24 genes involved in the biosynthetic toxin pathways were DEGs in these two strains. Among them, 16 were constitutively induced in the Ap2 MDR strain as compared to B05.10, while eight were suppressed (*XM_001555076.2*, *XM_024690498.1*, *XM_024691079.1*, *XM_001549738.2*, *XM_024693407.1*, *XM_024697593.1*, *XM_001545412.2* and *XM_024697594.1*) (Figure 2c).

### 2.3 Major transmembrane transporter genes induced in the MDR strain

**FIGURE 3:**
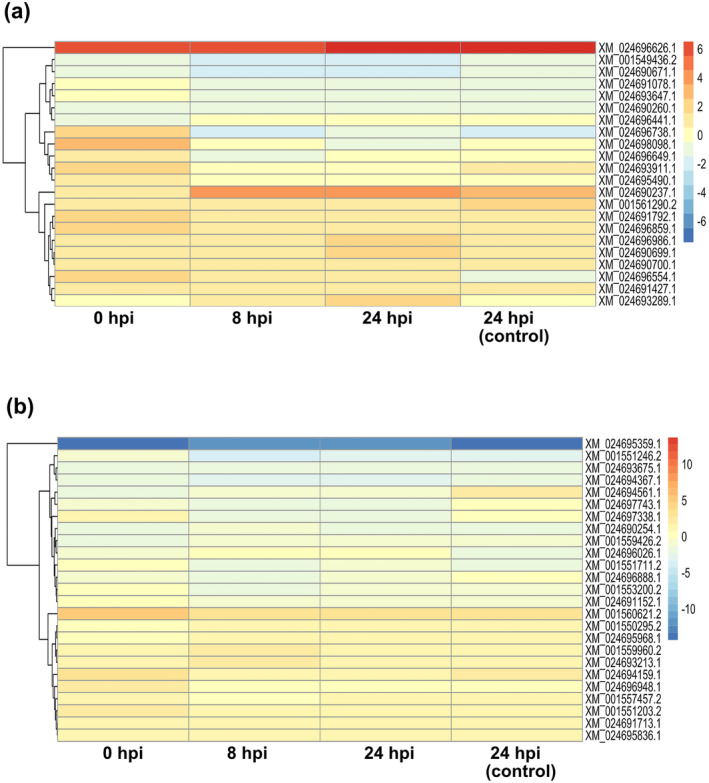
The *Botrytis cinerea* multidrug‐resistant Ap2 strain shows induction of the majority of genes encoding ABC and MFS transporters. (a) Differentially expressed genes encoding ABC transporters in Ap2 as compared to the B.05.10 strain. (b) Differentially expressed genes encoding MFS transporters, belong to the 2.A.1.3 family, in the Ap2 strain as compared to the B.05.10 strain. Analysis was conducted upon fungal exposure to the fungicide fludioxonil and the 0 and 24 h post‐inoculation (hpi)‐(control) samples represent no fungicide exposure and used as controls. (Adjusted *p*‐value <0.05, absolute log_2_ fold‐change >2 for up‐regulated genes and <−2 for the down‐regulated ones). Red and blue colours represent up‐regulated and down‐regulated genes, respectively.

As it is known that transmembrane transporter proteins are involved in fungicide multidrug resistance, we further focused our analysis on their expression patterns. We found 22 putative ABC transporter genes that were differentially expressed in Ap2 as compared to B05.10 (18 up‐regulated and 4 down‐regulated) even before exposure to fludioxonil (Figure 3a). Among them, the *BcatrB* (*XM_024696626*) and *BcatrO* (*XM_024690237*) genes were significantly induced in the resistant strain as compared to B05.10 in all studied conditions (Figure 3a). Eight hours after exposure to fludioxonil, the number of induced putative ABC transporter genes reduced to 19, while at 24 h after exposure the respective number of induced ABC transporter genes was 20 (Figure 3a). A phylogenetic analysis was conducted in order to categorize the differentially expressed ABC transporter genes in the different groups. Our analysis showed that the highly induced *BcatrB* gene and four other DEGs belonged to the G group, referred to as PDR (Figure S3) (Kovalchuck & Driessen, 2010). Regarding the rest of the differentially expressed ABC transporter genes, one was categorized in A group, seven in B group, four in C group, one in F group, while no clear classification was observed for two of them (Figure S3).

Regarding the MFS transporters, more than 100 genes were found to be differentially expressed in the Ap2 MDR strain in comparison to the B05.10 one (Figure S4). However, we focused our analysis on MFS transporters belonging to the 2.A.1.3 family (Drug:H + Antiporter‐2 (14 Spanner) [DHA2]), because this family refers to multidrug resistance (Paulsen et al., 1996; Reddy et al., 2012). Our studies showed that 24 genes belonging to this family were induced in Ap2 as compared to B05.10, while one gene (XM_024695359.1) was shown to be suppressed in all tested conditions (Figure 3b), suggesting a possible involvement of these transporters in the Ap2 resistance phenotype.

### 2.4 The Bcmfs3 MFS transporter is involved in increased tolerance against fluopyram and boscalid

**FIGURE 4:**
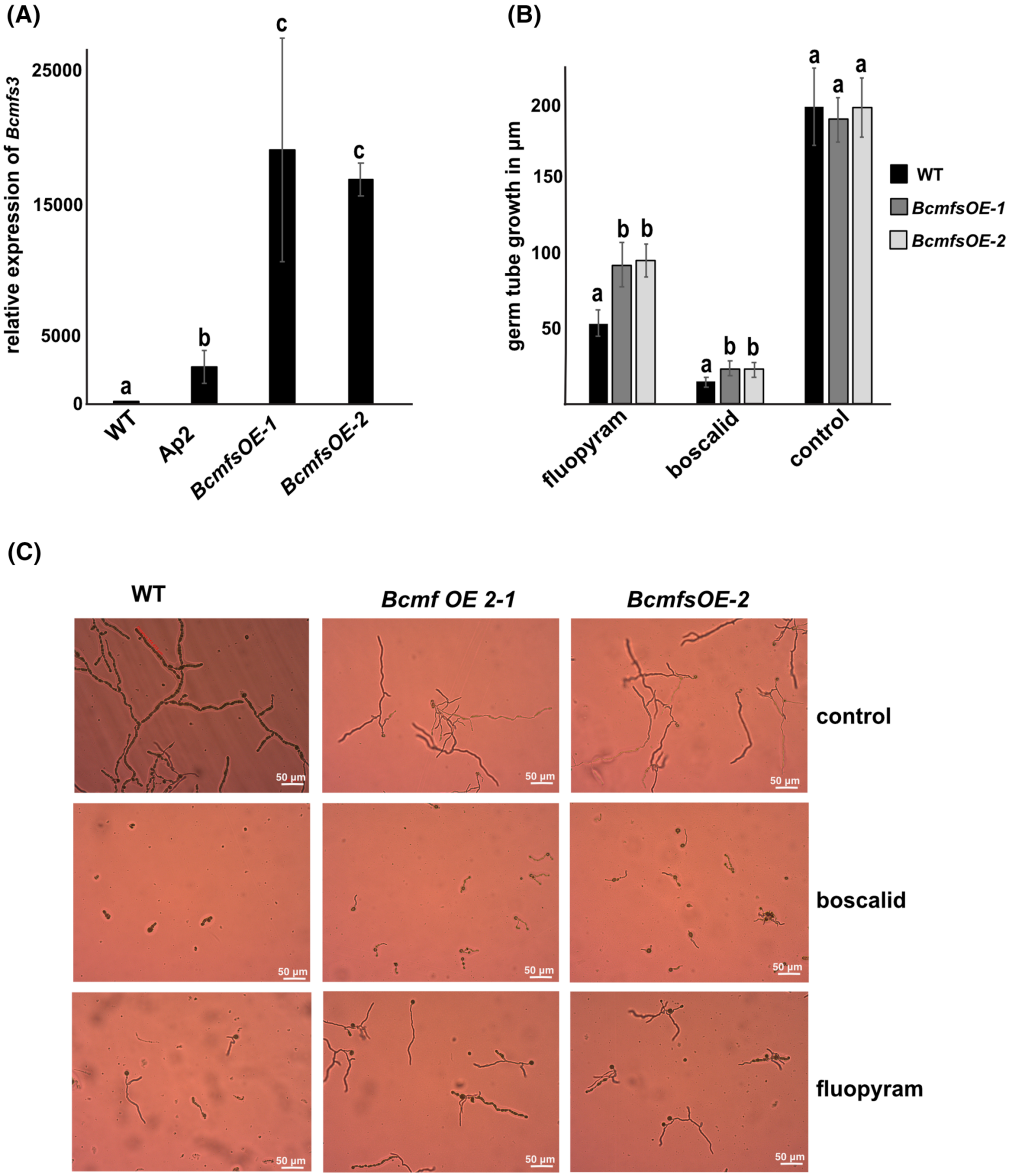
The Bcmfs3 MFS transporter increases tolerance to the fungicides boscalid and fluopyram. (A) Transcription analysis of *Botrytis cinerea* B05.10 strain overexpressing the *Bcmfs3* (*Bcmfs3‐OE*) gene in comparison to B05.10 wild type (WT) and to the Ap2 strain. Gene expression analysis was conducted according to the 2^−ΔΔ*C*t^ method. Data were normalized using the expression levels of the reference ubiquitin‐conjugating enzyme (*UCE*) gene. Different letters (a–c) indicate statistically significant differences according to Student's *t* test. Error bars represent *SE* based on three biological replicates. (B) Germ tube growth of *B. cinerea* *Bcmfs3*‐overexpression strains (*Bcmfs3 OE*), as compared to WT, upon exposure to either boscalid (0.05 ppm) or fluopyram (0.01 ppm). Growth in the absence of fungicide was used as a control. Different letters (a, b) indicate statistically significant differences according to Student's *t* test (*p* < 0.05). Error bars represent *SE* based on at least eight biological replicates. (C) Representative microscopic images showing the germ tube growth of *Bcmfs3 OE* and WT strains upon exposure to boscalid (0.05 ppm) and fluopyram (0.01 ppm). Non‐exposure to the fungicides was used as a control.

Among the 25 MFS transporter genes in the 2.A.1.3 family up‐regulated in Ap2, the *BCIN_03g04400* (*XM_001560621.2*) (*XP_001560671.2*) gene, denominated as *Bcmfs‐3*, was selected for further investigation because it showed high induction in all tested conditions. To determine its role in fungicide sensitivity levels, the gene was amplified from Ap2‐derived cDNA, ligated to a vector driven by the *gpdA* constitutively expressed promoter and transformed into the B05.10 strain. Two transformants with high expression of the *Bcmfs‐3* gene were identified by transcription analysis and so were selected for further analysis (Figure 4A). To investigate whether overexpression of this MFS transporter gene affects the mycelial growth, these transformants were inoculated in media with different concentrations of carbon sources. Our results showed that the *Bcmfs‐3OE* overexpression strains grew equally well as the wild type (WT) in all tested conditions (Figure S5), indicating that constitutive induction of this gene possibly does not have any serious phenotypic impact.

Further, their mycelial growth was tested in a variety of fungicides with different modes of action. We observed that the germ tubes of the MFS overexpression strains (*Bcmfs‐3OE*) showed a significantly (*p* = 0.0001) higher growth rate as compared to the WT strain in the presence of the SDHI fungicides fluopyram (0.01 ppm) and boscalid (0.05 ppm) at all tested concentrations (Figure 4B,C). The EC_50_ for these overexpression strains was calculated as 0.098 μg/mL for boscalid and 0.087 μg/mL for fluopyram (Table S2). For the reference B05.10 strain, the EC_50_ was calculated as 0.062 μg/mL and 0.031 μg/mL, respectively (Table S2). In contrast, no difference in mycelial growth between the *Bcmfs‐3 OE* and WT was observed when they were grown in the presence of pyraclostrobin (*Bcmfs‐3 OE* EC_50_ = 0.046/WT EC_50_ = 0.037), fludioxonil (*Bcmfs‐3 OE* EC_50_ = 0.009/WT EC_50_ = 0.008), fenhexamid (*Bcmfs‐3 OE* EC_50_ = 0.021/WT EC_50_ = 0.017), iprodione (*Bcmfs‐3 OE* EC_50_ = 0.89/WT EC_50_ = 0.78), cyprodinil (*Bcmfs‐3 OE* EC_50_ = 0.44/WT EC_50_ = 0.39), and tolnaftate (*Bcmfs‐3 OE* EC_50_ = 0.78/WT EC_50_ = 0.72), suggesting an involvement of this MFS transporter in efflux of SDHIs.

Finally, we investigated the expression patterns of *Bcmfs‐3* in five additional MDR strains. We found that this gene was constitutively expressed only in one strain as compared to B05.10 (Figure S6a), suggesting that possibly it is not a common MDR feature. However, induction of *Bcmfs‐3* was observed in the majority of the tested MDR strains (*p* = 0.012) (Figure S6b) in the presence of fluopyram, further supporting the role of this transporter to detoxification of SDHIs.

## 3 DISCUSSION

In the current study, a transcriptomic analysis of an MDR *B. cinerea* field strain was conducted, in order to elucidate the molecular mechanisms of this feature. The Ap2 strain that was used in this study had been isolated from peach rootstock seedling plants where fungicides had been applied extensively. As was expected, the development of strong resistance against a number of fungicides with diverse modes of action has already been observed (Sofianos et al., 2023). Analysis was performed upon exposure to fludioxonil at two different time points, and at two additional non‐exposure time points. This fungicide was chosen because moderate resistance has been observed and point mutations to the target genes are extremely rare, making it a robust marker for identification of MDR strains (Cosseboom et al., 2019; Rupp et al., 2017). We found that the total transcriptome of the MDR Ap2 strain differed significantly from the reference B05.10 one, something that was expected because these two strains are derived from different populations.

It is known that certain ABC and MFS transporter proteins are involved in fungicide multidrug resistance (Coleman & Mylonakis, 2009; Dos Santos et al., 2014). Because MDR‐type fungicide resistance is mainly attributed to high expression and/or high gene copy numbers of these transporters (Karlsson et al., 2015; Nygren et al., 2018), we first investigated their transcription patterns. Among the DEGs, ABC transporter genes the *BcatrB* and *BcatrO* genes were significantly induced in the Ap2 strain. These two ABC transporters have previously been reported to be involved in resistance to a variety of fungicides, and to the phytoalexin resveratrol as well (Hayashi et al., 2002b; Liu et al., 2023; Schoonbeek et al., 2002, 2003; Stefanato et al., 2009).

Regarding the MFS transporters, we identified more than 20 genes belonging to the 2.A.1.3 family that were induced in the MDR Ap2 strain. Previous data have shown the involvement of these transporters in fungicide resistance and in efflux of natural toxic compounds from fungal cells (Liu et al., 2023; Nygren et al., 2018; Samaras et al., 2020, 2021). High constitutive expression of certain MFS transporter genes in the Ap2 strain is in agreement with previous data from an MDR‐resistant *Penicillium expansum* strain (Samaras et al., 2020), and it is probably related to mutations in the transcription factors and/or the promoter of the target genes. Among these MFS transporters, we identified the *Bcmfs3* one to be highly induced in all tested conditions, and thus was selected for further analysis. We found that overexpression of this gene in the B05.10 reference strain led to an enhanced tolerance only against the fungicides boscalid and fluopyram, suggesting that *Bcmfs3* is specific to the SDHIs. This is in line with previous studies claiming that these transporters are more substrate‐specific than the ABC ones (Dos Santos et al., 2014; Zhang, Zhao, et al., 2015). While data regarding this described MFS transporter is rather limited, it seems that constitutive expression of this gene is not a common phenomenon. However, it seems to be induced in certain field strains in the presence of fluopyram. This could partly explain the fact that some field strains from heavily treated fields can be rather tolerant against fluopyram but at the same time not harbour any target site mutations that can be correlated with fluopyram resistance. Moreover, it is highly possible that more transporters could play a role in enhancing a strain tolerance to SDHIs and the combination of target site mutations along with MDR could further reduce fungicide sensitivity and at the same time widen the fungicide resistance profile of *B. cinerea* strains (Kretschmer et al., 2009; Leroch et al., 2011; Sofianos et al., 2023).

Fitness is defined as the ability of an organism to survive in its habitat and to reproduce successfully (Orr, 2009). Although it is known that in some cases fungicide resistance development is associated with fitness cost (Billard et al., 2011; Chen et al., 2022; Lalève et al., 2014; Veloukas et al., 2014), the exact mechanisms behind this phenomenon still remain widely unknown. More specifically, it is still unclear whether MDR phenotypes entail fitness costs and to what extent. Some studies suggest that the overexpression of these transporters can lead to enhanced traits because they could possibly detoxify the cell of secondary metabolites in addition to fungicides (Kretschmer et al., 2009). Nonetheless, it can be difficult to correlate MDR and fitness because most MDR strains also possess target site mutations in their genome as a result of the stepwise accumulation of resistance (Chen et al., 2016; Sofianos et al., 2023).

We showed that the Ap2 MDR strain showed faster growth on minimal media and a significant induction of a plethora of genes putatively encoding glucose transporters. These transporters also belong to the MFS family, and they are responsible for the extracellular uptake of glucose across the plasma membranes, a major step in glucose metabolism (Zhang, Cao, et al., 2015). Thus, better adaptation of this strain to carbon‐limited conditions is possibly related to up‐regulation of these transporter genes. Furthermore, a number of genes involved in toxin biosynthetic pathways were also induced in the MDR strain. Our data are in alignment with previous published studies showing that MDR occurred in the field, and could stimulate the production of secondary metabolites, possibly through induction of ABC transporters (Wu et al., 2024).

In this study we observed that the MDR field strain showed a reduced virulence as compared to the reference B05.10 strain. Our analysis also showed lower expression of genes in the MDR strain with involvement in *B. cinerea* virulence, and probably explains this phenotype. Among them, the *Bcbot1* gene has a confirmed role in the sesquiterpene botrydial biosynthesis pathway (Siewers et al., 2005). The botrydial biosynthetic gene cluster consists of five genes (*Bcbot1* to *Bcbot5*) that are co‐regulated by the Ca^2+^/calcineurin signal transduction pathway. The *Bcbot1* protein seems to be involved in a later step of botrydial biosynthesis (Siewers et al., 2005). It is produced during plant infection and causes chlorosis and death of cells, although the exact mechanism of phytotoxicity is not yet clear (Colmenares et al., 2002). However, its involvement in fungal virulence seems to be strain‐dependent (Siewers et al., 2005). Two genes encoding PKSs involved in the biosynthesis of the phytotoxin botcinic acid, which causes chlorotic and necrotic lesions in host plants (Cutler et al., 1996; Dalmais et al., 2011), were also suppressed in the Ap2 strain. Strains that produce both these phytotoxins are more virulent than strains that produce only botrydial (Reino et al., 2004). In addition, *BcOs1* appears to play a role in virulence too, because mutation of this gene in B05.10 led to the inability of *B. cinerea* to penetrate the host cells (Williamson et al., 2007), whereas a normal penetration was observed in the UWS 111 strain, indicating host‐dependence similar to botrydial (Viaud et al., 2006). In contrast, up‐regulation of the pectinase‐encoding gene *Bcpg2* was observed in the Ap2 field strain. Pectinases are thought to play an important role in fungal virulence, especially at the early stages of infection, when they are secreted together with effectors able to inactivate the polygalacturonase‐inhibiting proteins (Kars et al., 2005; Oeser et al., 2002; Wei et al., 2022). However, the necrotizing activity of polygalacturonases seems to be accession‐dependent in *A. thaliana* (Kars et al., 2005); thus, we could speculate that this gene probably plays a minor role in our studied pathosystem.

In conclusion, in this study we conducted transcriptomic and functional analyses in an MDR *B. cinerea* field strain. We found that induction of genes coding for ABC and MFS transporters pre‐exists in this strain, and we identified an MFS transporter that is probably involved in enhanced tolerance against the SDHI fungicides. Furthermore, we showed that this strain showed reduced virulence, with down‐regulation of genes with a confirmed role in fungal pathogenicity. The results from this study give insight towards a better understanding of MDR and the molecular mechanisms behind this, contributing to the development of more efficient control strategies.

## 4 EXPERIMENTAL PROCEDURES

### 4.1 Fungal strains and growth conditions

In the current study, the *B. cinerea* reference strain B05.10 and a set of six field strains exhibiting the MDR phenotype were used. Field strains were collected and characterized for the requirements of a recent monitoring programme aiming to determine the status of *B. cinerea* populations' sensitivity to fungicides in Greece (Sofianos et al., 2023). Details on the fungicide sensitivity and the genotypic characteristics of the strains used in the study are provided in Table S2. Among them, the Ap2 strain was selected for further analysis because it was resistant to all available botryticides. The resistance of Ap2 strain to fungicides was associated with target site mutations (H272R in SdhB, L412F in Bcpos5 and F412S in Erg27) and the ΔL/V497 in *mrr1* gene associated with the overexpression of *atrB* and the MDR phenotype of the strain (Table S2).

### 4.2 Nucleic acid manipulations and transcriptomic analysis

For the transcriptomic analysis, mycelia from the B05.10 and Ap2 strains were cultured in potato dextrose broth (PDB; Difco). After 2 days of incubation at 20°C, the B05.10 and Ap2 cultures were amended with 0.005 and 0.2 μg/mL fludioxonil, respectively. Because the sensitivity of these two strains to fludioxonil was different, the applied concentration was calculated based on their EC_50_. The mycelia were collected at zero (no exposure control), 8 and 24 h after exposure. A 24 h non‐exposure control was also used. Total RNA was extracted from the collected mycelia using the Spectrum Plant Total RNA Kit (Sigma‐Aldrich), according to the manufacturer's instructions, and 1 μg of total RNA were treated with DNase I (Thermo Fisher Scientific). Afterwards, RNA strand‐specific libraries were generated using the TruSeq stranded mRNA library preparation kit with polyA selection (Illumina, Inc.). RNA strand‐specific libraries were sequenced using Illumina NovaSeq 6000 at the SNP&SEQ Technology Platform, Science for Life Laboratory at Uppsala University, Sweden. Four biological replicates per treatment were used.

For the bioinformatic analysis quality‐trimming and adapter removal were conducted with bbduk v. 38.9 (Bushnell, 2018), the fastqc v. 11.9 quality control tool was used to verify the quality of the clean reads (Andrews, 2010). Reads were then mapped to the *B. cinerea* B05.10 genome (GenBank assembly accession: GCA_000143535.4) using the splice‐aware aligner STAR v. 2.7.9a (Dobin et al., 2013). The number of reads mapping to each transcript was quantified using featureCounts v. 2.0.1 (Liao et al., 2014), and differential expression was determined with the DESeq2 R package v. 1.28.1 (Love et al., 2014) using a minimal threshold of 1 for log_2_(fold‐change) and 0.05 for false discovery rate (FDR) adjusted *p*‐value. Data visualization was performed with the R pheatmap module (Kolde & Kolde, 2015). The *B. cinerea* B05.10 proteome, retrieved from NCBI, was compared to the PHI‐base database v. 09‐05‐2022 using BLASTP (Ye et al., 2006) with minimum 80% in both identity and query coverage to determine known or putative factors of virulence (Urban et al., 2017).

### 4.3 Mycelial growth and virulence assays

The mycelial growth of Ap2 and B05.10 strains was tested on four different growth media: (a) minimal medium (MM; 1 g KH_2_PO_4_, 0.5 g MgSO_4_, 0.5 g KCl, 1 mg FeSO_4_.7H_2_O, 20 g glucose, 2 g NaNO_3_, 1.5% agar in 1 L deionized water); (b) intermediate nutrient medium (IM; 4 g peptone, 4 g glucose, 0.75 g KH_2_PO_4_, 2 g MgSO_4_.7H_2_O, 2 g citric acid, 0.02% Tween 20, 1.5% agar in 1 L deionized water adjusted to pH 5.6); (c) carbon‐rich V8 medium (3 g CaCO_3_, 28 g KCl, 200 mL V8 [Granini], 1.5% agar in 1 L deionized water), and (d) potato dextrose agar (PDA; Difco). For the plant virulence assays, rosette leaves of 4‐week‐old *A. thaliana* plants grown under short‐day conditions (8 h light/16 h dark) at 22°C/17°C were used. For inoculum production, the two strains were grown on hydroxyapatite (HA) medium (4 g glucose, 4 g yeast extract, 10 g malt extract, 1.5% agar, 1 L deionized water) and incubated for 14 days at 20°C. Spore suspensions were prepared in Gamborg's B5 minimal medium (3 g Gamborg B5 basal salt mixtures, 1.36 g KH_2_PO_4_, and 9.9 g glucose, in 1 L deionized water) and adjusted to a concentration of 10^5^ spores/mL. Then, 10 μL of this suspension was placed on the adaxial side of each leaf. Control plants were inoculated with 10 μL of deionized water, and symptoms were evaluated after 3 days. The lesion area was calculated using the ImageJ software (National Institutes of Health). Four biological replicates were used, and each sample comprised four plants and three or four infected leaves per plant.

### 4.4 Phylogenetic analysis and classification of MFS transporters

Phylogenetic analysis of differentially regulated ABC‐transporter genes in *B. cinerea* strains and homologues from selected fungal species was conducted based on amino acid sequence alignments created with the CLUSTALW algorithm. The analysis was carried out using the neighbour‐joining (NJ) method implemented in the MEGA v. 7 software (Kumar et al., 2018), using the JTT substitution model (Jones et al., 1992) using all sites and 1000 bootstrap replicates. Categorization of MFS transporter gene families was conducted according to the Transporter Classification Database (Saier et al., 2016).

### 4.5 Overexpression vector and fungal transformation

For overexpression of the *Bcmfs3* gene, the GeneArt Seamless cloning technology (Invitrogen) was used. Briefly, the *Bcmfs3* gene derived from Ap2 cDNA, and the pRFHUE‐eGFP vector (Crespo‐Sempere et al., 2011), driven by the *gpdA* constitutively expressed promoter, was amplified using the primers listed in Table S3, designed by the GeneArt software (Invitrogen). PCRs were carried out using the Phusion Green Hot Start II High‐Fidelity PCR master mix (Thermo Scientific), and the correctly assembled vectors were confirmed by restriction enzyme digestion analysis and Sanger sequencing (Macrogen).

For fungal transformation in the B05.10 strain, a protoplast‐based protocol was used (Müller et al., 2018). Four to five days later, positive colonies were transferred on HA plates containing the appropriate concentration of hygromycin (17.5 μg/mL). Mitotically stable transformants were subcultured on selective medium containing 35 μg/mL hygromycin and validated using reverse transcription‐quantitative PCR (RT‐qPCR) techniques. Briefly, 1 μg of total DNase‐treated RNA was reverse transcribed using iScript cDNA synthesis kit (Bio‐Rad). RT‐qPCR analysis was conducted on CFX Connect Real‐Time PCR Detection System (Bio‐Rad) using primers listed in Table S3. Expression of genes was normalized using the expression levels of the ubiquitin‐conjugating enzyme (*UCE*) gene (Ren et al., 2017). Relative expression values were calculated from the threshold cycle (*C*_t_) values according to the 2^−ΔΔ*C*t^ method (Livak & Schmittgen, 2001). Three biological replicates per mutant and control were used, including two technical replicates for each biological one.

### 4.6 Fungicide sensitivity assays

The sensitivity of overexpression transformants (*Bcmfs3_ΟΕ*) to several botryticides was measured in terms of EC_50_ values following procedures described previously (Konstantinou et al., 2015; Sofianos et al., 2023). Sensitivity to the SDHIs fluopyram and boscalid was monitored on yeast Bacto acetate agar (YBA) medium (10 g yeast extract, 10 g peptone, 20 g sodium acetate, 15 g agar in 1 L distilled water), to pyraclostrobin, fludioxonil, fenhexamid and iprodione was monitored on HA medium and to cyprodinil was monitored on Gamborg's B5 minimal medium amended with different doses of the respective fungicides. Pyraclostrobin‐amended media were also amended with 100 mg/L of salicylic hydroxamic acid (SHAM; Sigma) to prevent alternative respiration. The EC_50_ values were determined by plotting the relative inhibition of the diameter of mycelial growth or the relative inhibition of germ tube growth against the log_10_ of fungicide concentrations. Calculations were performed with the use of SAS (JMP; SAS Institute). Resistance factors (RFs) were calculated by dividing the EC_50_ value of each strain with the EC_50_ value of the reference strain B05.10.

### 4.7 *Bcmfs3* transcription analysis in field MDR strains

Transcription analysis of the *Bcmfs3* was conducted in six MDR *B. cinerea* strains (Table S2) in the presence (0.01 ppm) or absence of fluopyram. RNA extraction, cDNA synthesis and RT‐qPCR analysis were carried out as described above. Three biological replicates per mutant and control were used, including two technical replicates for each biological one.

### 4.8 Statistical analysis

Analysis of variance (ANOVA, one‐way) was conducted on gene expression and phenotypic data using a general linear model implemented in SPSS v. 24 (IBM). Pairwise comparisons were performed using the Student's *t* test at the 95% significance level.

## CONFLICT OF INTEREST STATEMENT

The authors declare that there are no conflicts of interest.

## Supporting information


**FIGURE S1.** GO analysis in biological process between *Botrytis cinerea* B05.10 and Ap2 strains during 0 h post‐inoculation exposure to fludioxonil (no exposure). The symbol > shows up‐regulation.

**FIGURE S2.** Phenotypic analysis of *Botrytis cinerea* Ap2 MDR and B05.10 strains. (a) Mycelial growth in cm of Ap2 and B05.10, 5 days post‐inoculation (dpi). (b) Lesion area on *Arabidopsis thaliana* leaves infected with *B. cinerea* Ap2 and B05.10 strains 3 dpi. Asterisks (*) represent statistically significant differences according to Student’s *t* test (*p* < 0.05). Error bars represent *SE* based on five biological replicates. (c) Symptoms on *A. thaliana* leaves infected by the Ap2 and B.05.10 strains. Photographs of representative plants were taken 3 dpi.

**FIGURE S3.** Phylogenetic analysis of ABC transporters induced in *Botrytis cinerea* Ap2 strain. Analysis was conducted using the maximum likelihood with the JTT amino acid substitution model based on amino acid sequences and 500 bootstraps. Number at nodes indicate the bootstrap values. Bar indicates the number of amino acid substitution. Predicted amino acidic sequences were aligned using the CLUSTALW algorithm and phylogeny was constructed in the MEGA X software using neighbour‐joining method. Bootstrap support values from 1000 iterations are associated with the nodes.

**FIGURE S4.** Heatmaps of (a) up‐regulated and (b) down‐regulated genes putatively encode MFS transporters in the Ap2 strain as compared to the B05.10 upon exposure to the fludioxonil fungicide. Data were normalized to the zero‐time point exposure (adjusted *p*‐value <0.05, absolute log_2_ fold‐change >2 for up‐regulated genes and < −2 for the down‐regulated ones). Yellow and blue colours represent up‐regulated and down‐regulated genes, respectively.

**FIGURE S5.** Mycelial growth of *Botrytis cinerea* Bcmfs3 overexpression strains, on different carbon sources; potato dextrose agar (PDA), V8, intermediate medium (IM) and minimal medium (MM), 5 days post‐inoculation.

**FIGURE S6.** Transcription analysis of Bcmfs3 in *Botrytis cinerea* multidrug resistant (MDR) strains comparing to B05.10, upon (a) the absence or (b) the presence of fluopyram 24 h post‐inoculation. Gene expression analysis was conducted according to 2^−ΔΔ*Ct*^ method. Data were normalized using the expression levels of the reference ubiquitin‐conjugating enzyme (*UCE*) gene. Asterisks (*) indicate statistically significant differences according to Student’s *t* test. Error bars represent *SE* based on three biological replicates.

**Table S1.** Annotation of genes used in the presented heatmaps.

**TABLE S2.** Sensitivity of *Botrytis cinerea* strains to the SDHI fungicides boscalid and fluopyram and target site mutations in *sdhB*, e*rg27*, *pos5* and *mrr1* genes.

**TABLE S3.** Primers used in the current study.

## Data Availability

The transcriptomic data for this study have been deposited in the European Nucleotide Archive (ENA) at EMBL‐EBI under Bioproject accession number PRJEB61110 (https://www.ebi.ac.uk/ena/browser/view/PRJEB61110).
