# Supplementary material for: Transcriptomic and functional analyses on a Botrytis cinerea multidrug‐resistant (MDR) strain provides new insights into the potential molecular mechanisms of MDR and fitness
Source: Mol Plant Pathol. 2024 Sep 7;25(9):e70004. doi: 10.1111/mpp.70004 (PMC11380696; doi:10.1111/mpp.70004)
Supplement: Supplementary file 1 — FIGURE S1. GO analysis in biological process between Botrytis cinerea B05.10 and Ap2 strains during 0 h post‐inoculation exposure to fludioxonil (no exposure). The symbol > shows up‐regulation. [file MPP-25-e70004-s001.pdf]

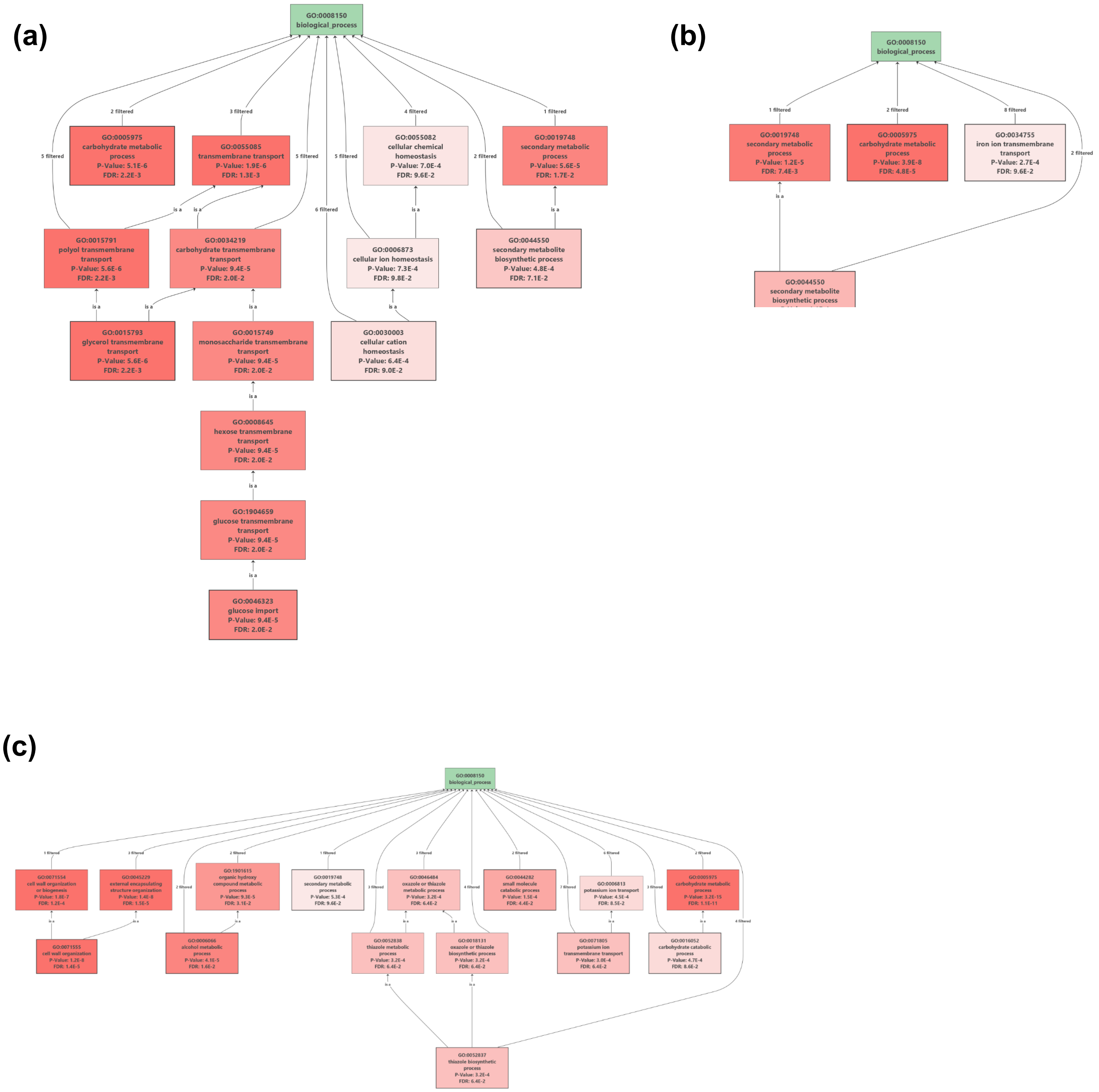
(a)
GO:0008150 biological_process
GO:0005975 carbohydrate metabolic process P-Value: 5.1E-6 FDR: 2.2E-3
GO:0055085 transmembrane transport P-Value: 1.9E-6 FDR: 1.3E-3
GO:0055082 cellular chemical homeostasis P-Value: 7.0E-4 FDR: 9.6E-2
GO:0019748 secondary metabolic process P-Value: 5.6E-5 FDR: 1.7E-2
GO:0015791 polyol transmembrane transport P-Value: 5.6E-6 FDR: 2.2E-3
GO:0034219 carbohydrate transmembrane transport P-Value: 9.4E-5 FDR: 2.0E-2
GO:0006873 cellular ion homeostasis P-Value: 7.3E-4 FDR: 9.8E-2
GO:0044550 secondary metabolite biosynthetic process P-Value: 4.8E-4 FDR: 7.1E-2
GO:0015793 glycerol transmembrane transport P-Value: 5.6E-6 FDR: 2.2E-3
GO:0015749 monosaccharide transmembrane transport P-Value: 9.4E-5 FDR: 2.0E-2
GO:0030003 cellular cation homeostasis P-Value: 6.4E-4 FDR: 9.0E-2
GO:0008645 hexose transmembrane transport P-Value: 9.4E-5 FDR: 2.0E-2
GO:1904659 glucose transmembrane transport P-Value: 9.4E-5 FDR: 2.0E-2
GO:0046323 glucose import P-Value: 9.4E-5 FDR: 2.0E-2
(b)
GO:0008150 biological_process
GO:0019748 secondary metabolic process P-Value: 1.2E-5 FDR: 7.4E-3
GO:0005975 carbohydrate metabolic process P-Value: 3.9E-8 FDR: 4.8E-5
GO:0034755 iron ion transmembrane transport P-Value: 2.7E-4 FDR: 9.6E-2
GO:0044550 secondary metabolite biosynthetic process
(c)
GO:0008150 biological_process
GO:0071554 cell wall organization or biogenesis P-Value: 1.8E-7 FDR: 1.2E-4
GO:0045229 external encapsulating structure organization P-Value: 1.4E-8 FDR: 1.5E-5
GO:1901615 organic hydroxy compound metabolic process P-Value: 9.3E-5 FDR: 3.1E-2
GO:0019748 secondary metabolic process P-Value: 5.3E-4 FDR: 9.6E-2
GO:0046484 oxazole or thiazole metabolic process P-Value: 3.2E-4 FDR: 6.4E-2
GO:0044282 small molecule catabolic process P-Value: 1.5E-4 FDR: 4.4E-2
GO:0006813 potassium ion transport P-Value: 4.5E-4 FDR: 8.5E-2
GO:0005975 carbohydrate metabolic process P-Value: 3.2E-15 FDR: 1.1E-11
GO:0071555 cell wall organization P-Value: 1.2E-8 FDR: 1.4E-5
GO:0006066 alcohol metabolic process P-Value: 4.1E-5 FDR: 1.6E-2
GO:0052838 thiazole metabolic process P-Value: 3.2E-4 FDR: 6.4E-2
GO:0018131 oxazole or thiazole biosynthetic process P-Value: 3.2E-4 FDR: 6.4E-2
GO:0071805 potassium ion transmembrane transport P-Value: 3.0E-4 FDR: 6.4E-2
GO:0016052 carbohydrate catabolic process P-Value: 4.7E-4 FDR: 8.6E-2
GO:0052837 thiazole biosynthetic process P-Value: 3.2E-4 FDR: 6.4E-2

Ap2 > B05.10

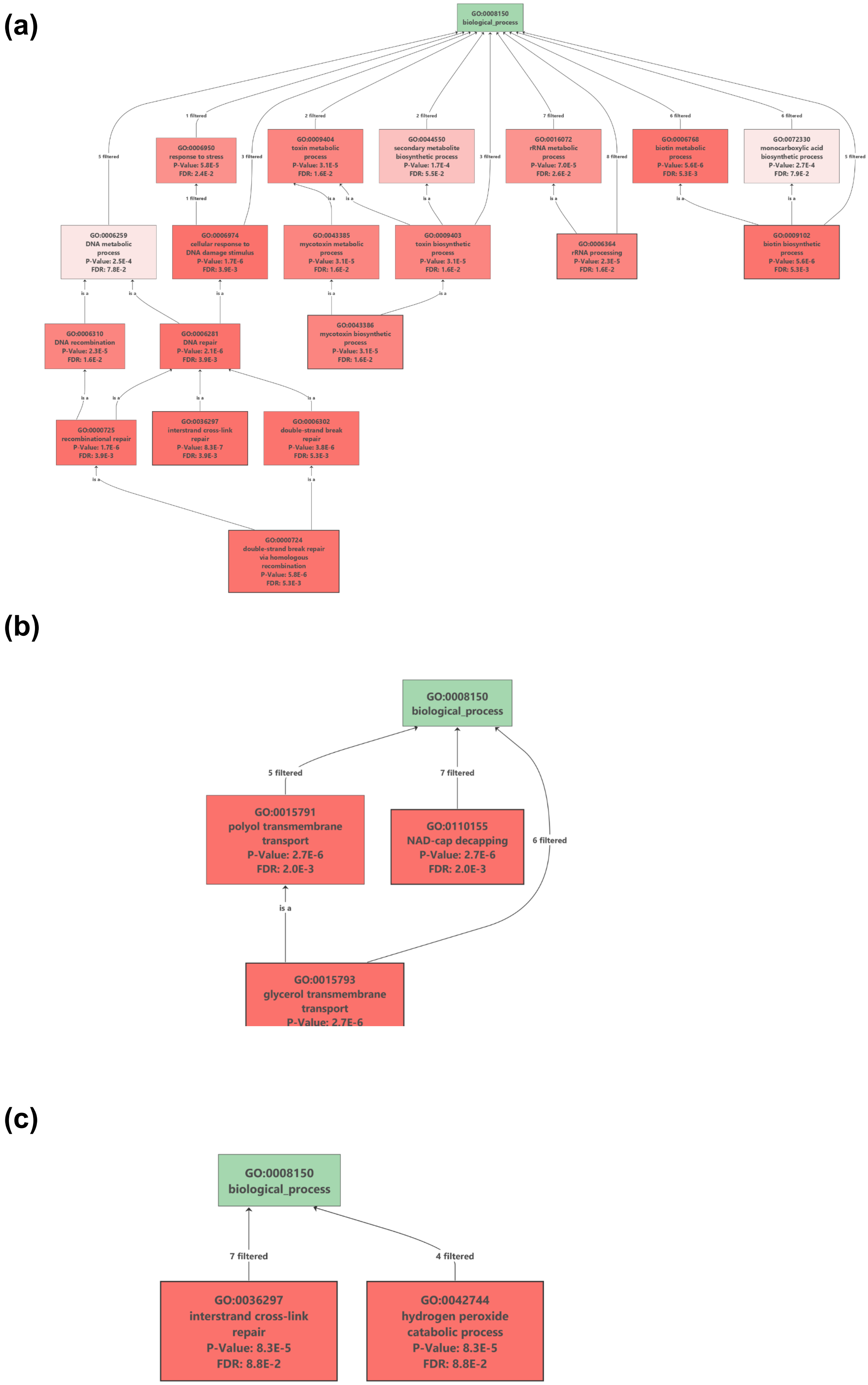

B05.10 > Ap2

Figure S1. GO analysis in biological process between *Botrytis cinerea* B05.10 and Ap2 isolates (a) 0 hpi (no exposure) (b) 8 hpi and (c) 24 hpi exposure to fludioxonil. The symbol ">" shows up-regulation.
