# Supplementary material for: Transcriptomic and functional analyses on a Botrytis cinerea multidrug‐resistant (MDR) strain provides new insights into the potential molecular mechanisms of MDR and fitness
Source: Mol Plant Pathol. 2024 Sep 7;25(9):e70004. doi: 10.1111/mpp.70004 (PMC11380696; doi:10.1111/mpp.70004)
Supplement: Supplementary file 2 — FIGURE S2. Phenotypic analysis of Botrytis cinerea Ap2 MDR and B05.10 strains. (a) Mycelial growth in cm of Ap2 and B05.10, 5 days post‐inoculation (dpi). (b) Lesion area on Arabidopsis thaliana leaves infected with B. cinerea Ap2 and B05.10 strains 3 dpi. Asterisks (*) represent statistically significant differences according to Student’s t test (p < 0.05). Error bars represent SE based on five biological replicates. (c) Symptoms on A. thaliana leaves infected by the Ap2 and B.05.10 strains. Photographs of representative plants were taken 3 dpi. [file MPP-25-e70004-s003.pdf]

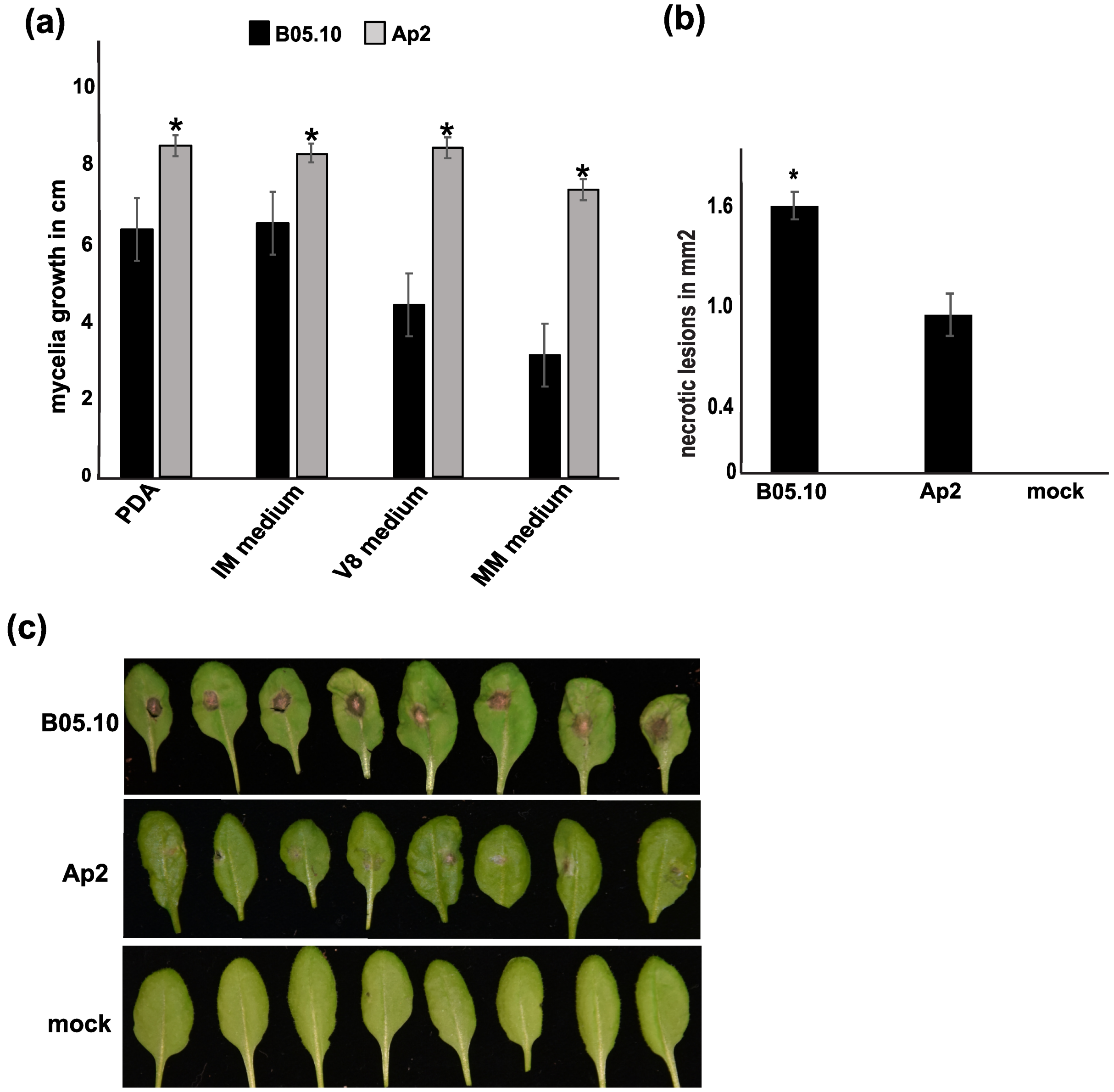

Figure S2. Phenotypic analysis of *B. cinerea* Ap2 MDR and B05.10 strains. (a) Mycelia growth in cm of Ap2 and B05.10, on media with different concentrations in sucrose; rich (V8), intermediate (IM) and minimal (MM) five dpi. (b) Lesion area on *A. thaliana* leaves infected with *B. cinerea* Ap2 and B05.10 isolates three dpi. (c) Symptoms on *A. thaliana* leaves infected by the Ap2 and B.05.10 isolates. Photographs of representative plants were taken three dpi. Asterisks (*) represent statistically significant differences according to the Student's T test ($p<0.05$). Error bars represent SE based on five biological replicates.
