# Supplementary material for: Transcriptomic and functional analyses on a Botrytis cinerea multidrug‐resistant (MDR) strain provides new insights into the potential molecular mechanisms of MDR and fitness
Source: Mol Plant Pathol. 2024 Sep 7;25(9):e70004. doi: 10.1111/mpp.70004 (PMC11380696; doi:10.1111/mpp.70004)
Supplement: Supplementary file 3 — FIGURE S3. Phylogenetic analysis of ABC transporters induced in Botrytis cinerea Ap2 strain. Analysis was conducted using the maximum likelihood with the JTT amino acid substitution model based on amino acid sequences and 500 bootstraps. Number at nodes indicate the bootstrap values. Bar indicates the number of amino acid substitution. Predicted amino acidic sequences were aligned using the CLUSTALW algorithm and phylogeny was constructed in the MEGA X software using neighbour‐joining method. Bootstrap support values from 1000 iterations are associated with the nodes. [file MPP-25-e70004-s006.pdf]

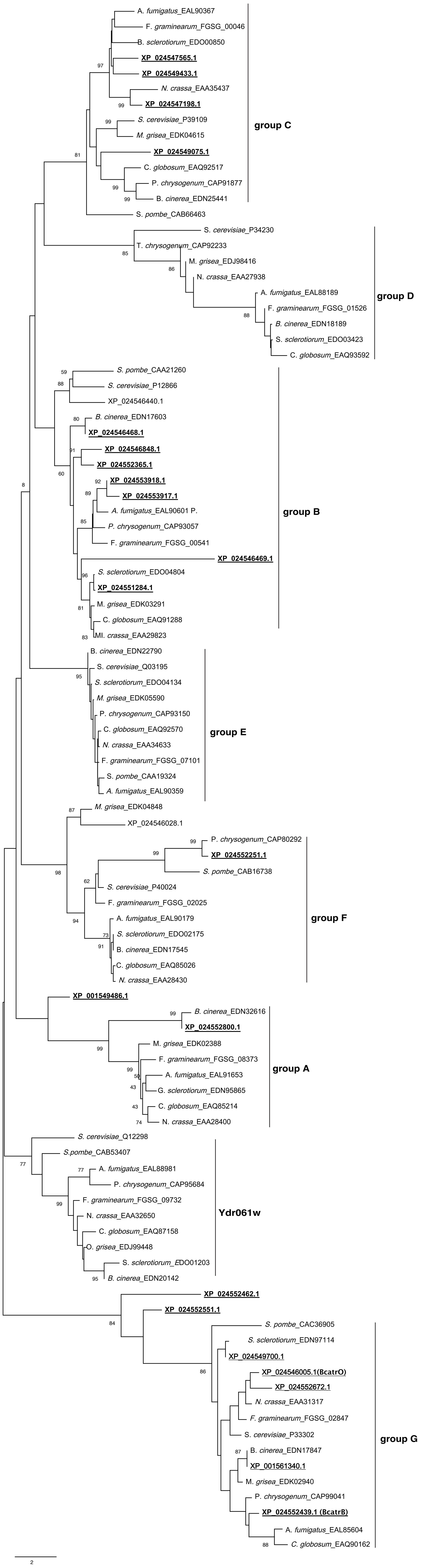

Figure S3. Phylogenetic analysis of ABC transporters induced in *B. cinerea* Ap2 isolate. Analysis was conducted using the maximum likelihood with the JTT amino acid substitution model based on amino acid sequences and 500 bootstraps. Number at nodes indicate the bootstrap values. Bar indicates the number of amino acid substitution. Predicted amino acidic sequences were aligned using the CLUSTAL W algorithm and phylogeny was constructed in the MEGA X software using neighbour-joining method. Bootstrap support values from 500 iterations are associated with the nodes
