# Supplementary material for: Transcriptomic and functional analyses on a Botrytis cinerea multidrug‐resistant (MDR) strain provides new insights into the potential molecular mechanisms of MDR and fitness
Source: Mol Plant Pathol. 2024 Sep 7;25(9):e70004. doi: 10.1111/mpp.70004 (PMC11380696; doi:10.1111/mpp.70004)
Supplement: Supplementary file 4 — FIGURE S4. Heatmaps of (a) up‐regulated and (b) down‐regulated genes putatively encode MFS transporters in the Ap2 strain as compared to the B05.10 upon exposure to the fludioxonil fungicide. Data were normalized to the zero‐time point exposure (adjusted p‐value <0.05, absolute log2 fold‐change >2 for up‐regulated genes and < −2 for the down‐regulated ones). Yellow and blue colours represent up‐regulated and down‐regulated genes, respectively. [file MPP-25-e70004-s004.pdf]

(a)

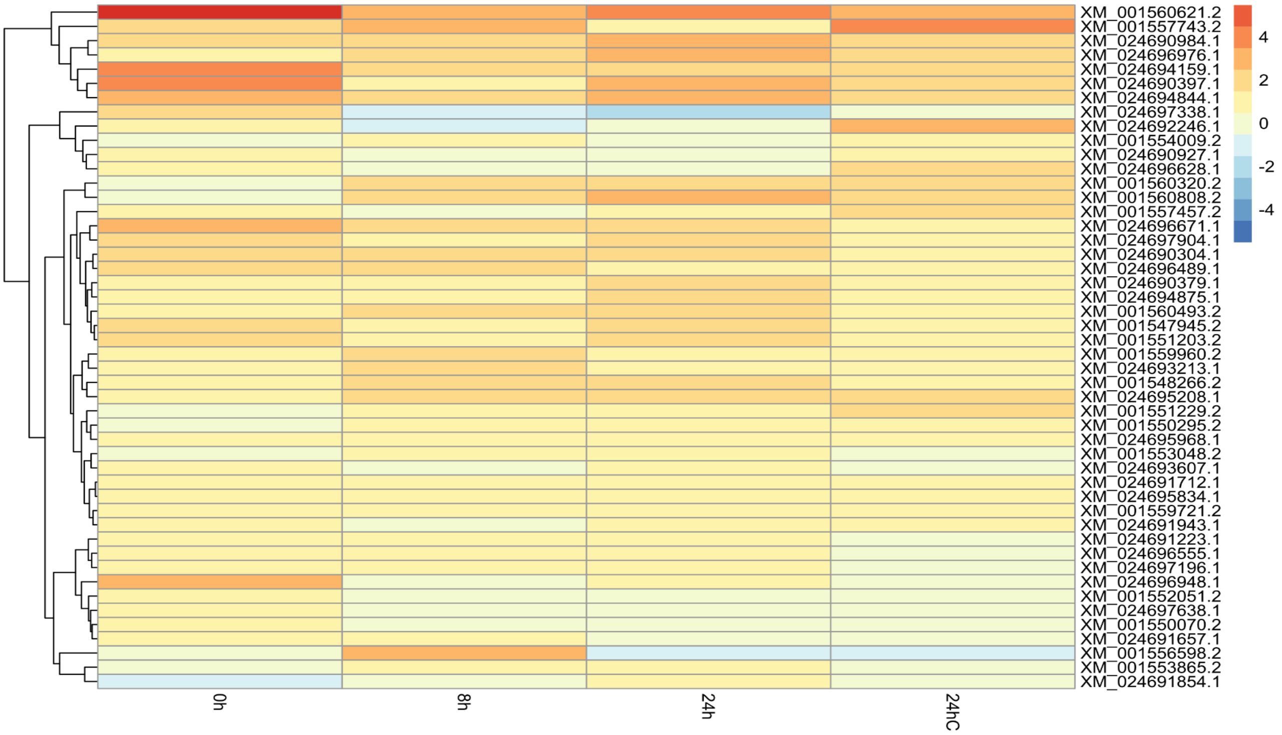

(b)

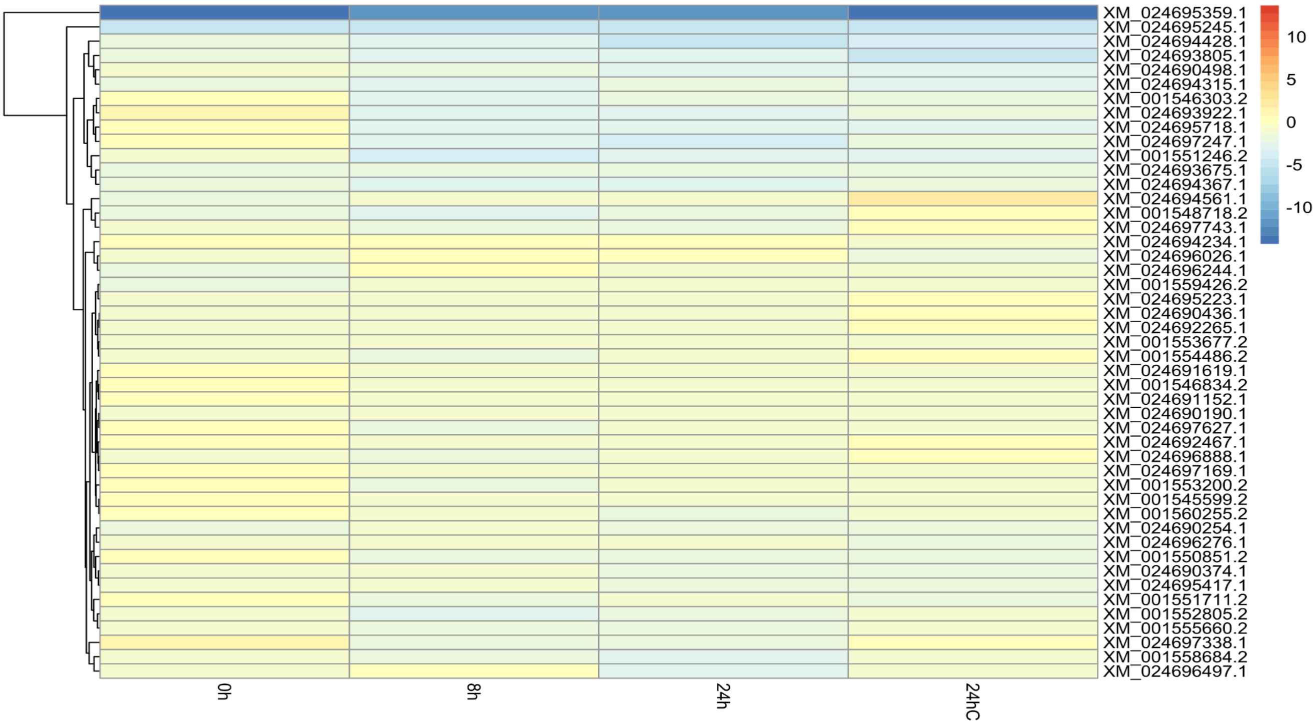

Figure S4. Heatmaps of (A) up-regulated and (B) down-regulated genes putatively encode mfs transporters in the Ap2 isolate as compared to the B05.10 upon exposure to the fludioxonil fungicide Data were normalized to the zero-time point exposure (adjusted p-value < 0.05, absolute log2 fold change > 2 for up-regulated genes and < -2 for the downregulated ones). Yellow and blue colors represent up-regulated or down-regulated genes respectively.
