# Supplementary material for: Transcriptomic and functional analyses on a Botrytis cinerea multidrug‐resistant (MDR) strain provides new insights into the potential molecular mechanisms of MDR and fitness
Source: Mol Plant Pathol. 2024 Sep 7;25(9):e70004. doi: 10.1111/mpp.70004 (PMC11380696; doi:10.1111/mpp.70004)
Supplement: Supplementary file 5 — FIGURE S5. Mycelial growth of Botrytis cinerea Bcmfs3 overexpression strains, on different carbon sources; potato dextrose agar (PDA), V8, intermediate medium (IM) and minimal medium (MM), 5 days post‐inoculation. [file MPP-25-e70004-s009.pdf]

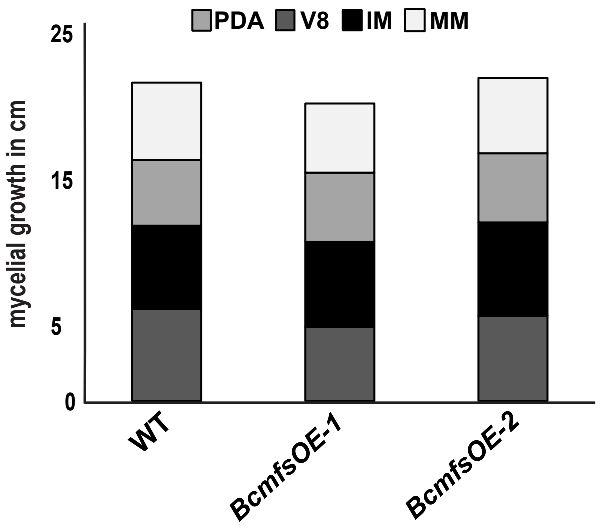

Figure S5. Mycelial growth of *B. cinerea* Bcmfs3 overexpression strains, on different carbon sources; PDA, V8, Intermediate media (IM) and minimal media (MM), 5 dpi.
