# Supplementary material for: Transcriptomic and functional analyses on a Botrytis cinerea multidrug‐resistant (MDR) strain provides new insights into the potential molecular mechanisms of MDR and fitness
Source: Mol Plant Pathol. 2024 Sep 7;25(9):e70004. doi: 10.1111/mpp.70004 (PMC11380696; doi:10.1111/mpp.70004)
Supplement: Supplementary file 6 — FIGURE S6. Transcription analysis of Bcmfs3 in Botrytis cinerea multidrug resistant (MDR) strains comparing to B05.10, upon (a) the absence or (b) the presence of fluopyram 24 h post‐inoculation. Gene expression analysis was conducted according to 2−ΔΔCt method. Data were normalized using the expression levels of the reference ubiquitin‐conjugating enzyme (UCE) gene. Asterisks (*) indicate statistically significant differences according to Student’s t test. Error bars represent SE based on three biological replicates. [file MPP-25-e70004-s002.pdf]

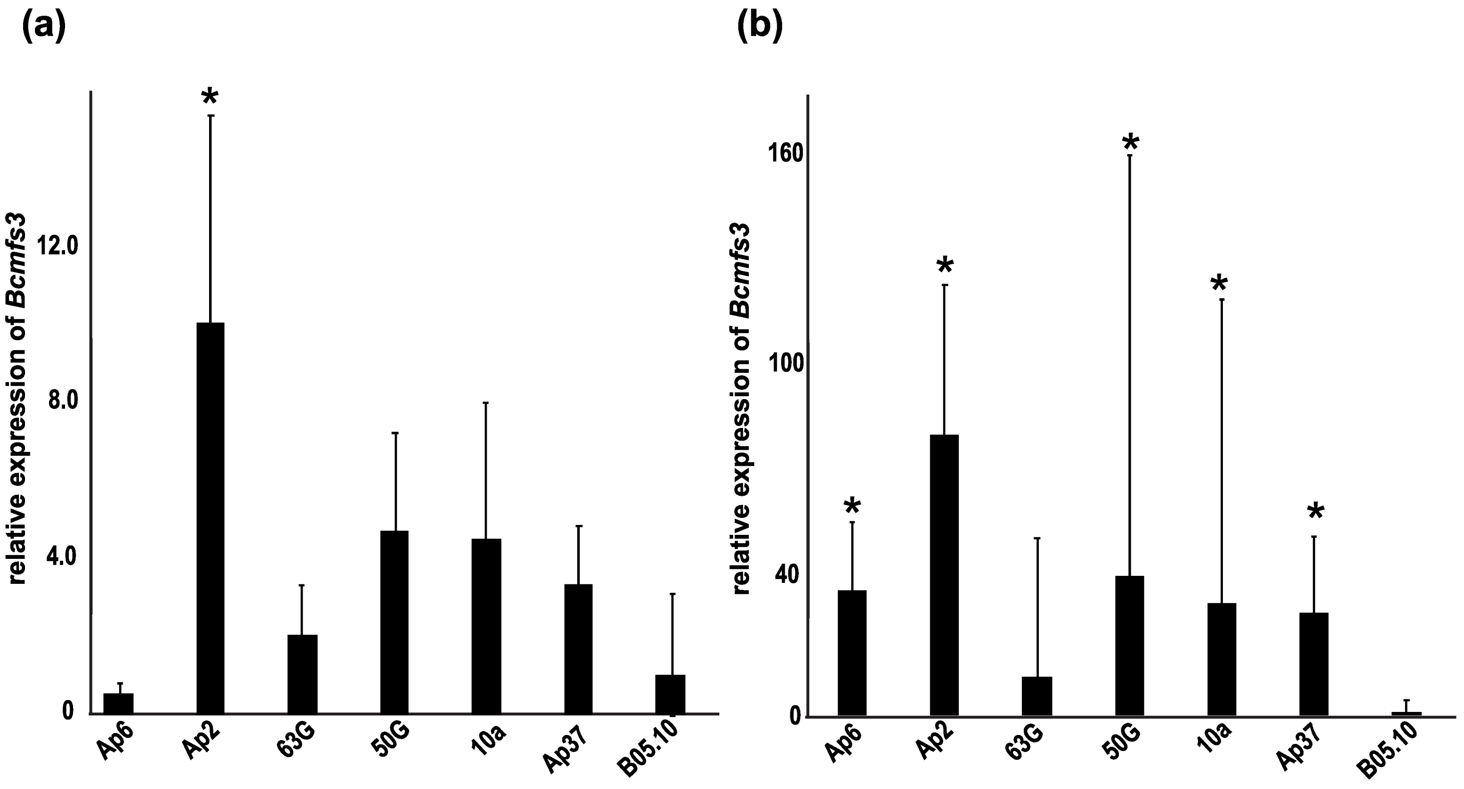

Figure S6. Transcription analysis of *Bcmfs3* in *B. cinerea* MDR isolates comparing to B05.10, upon (a) absence or (b) presence of fluopyram 24hpi. Gene expression analysis was conducted according to $2^{-\Delta\Delta CT}$ method. Data were normalized using the expression levels of the reference ubiquitin-conjugating enzyme *(UCE)* gene. Asterisks (*) indicate statistically significant differences according to the Students T test. Error bars represent standard deviation (SD) based on three biological replicates.
