# Supplementary material for: Transcriptomic and functional analyses on a Botrytis cinerea multidrug‐resistant (MDR) strain provides new insights into the potential molecular mechanisms of MDR and fitness
Source: Mol Plant Pathol. 2024 Sep 7;25(9):e70004. doi: 10.1111/mpp.70004 (PMC11380696; doi:10.1111/mpp.70004)
Supplement: Supplementary file 8 — TABLE S2. Sensitivity of Botrytis cinerea strains to the SDHI fungicides boscalid and fluopyram and target site mutations in sdhB, erg27, pos5 and mrr1 genes. [file MPP-25-e70004-s005.docx]

| Table S2. Sensitivity of *Botrytis cinerea* isolates to the SDHI fungicides boscalid and fluopyram and target site mutations in *sdh*B, e*rg27*, *pos5* and *mrr1* genes | | | | | | | | | | |
| --- | --- | --- | --- | --- | --- | --- | --- | --- | --- | --- |
| Mutations | | | | | | **Fungicide sensitivity** | | | | |
|  |  |  |  |  |  | boscalid | | | fluopyram | |
| Isolates | | *sdhB* | *erg27* | *pos5* | *mrr1* | EC_50_  (μg ml^-1^) | | RF^1^ | EC_50_  (μg ml^-1^) | RF |
| Ap2  Ap6  Ap37  10a  63G  50G | H272R  H272R  H272R  H272R  -  - | | F412S  F412S  T63I  -  -  F412S | L412F  L412F  L412V  -  F412F  - | ΔL/V497  ΔL/V497  ΔL/V497  -  V575G  - | | 4.4  4.1  6.64  2.41  0.069  0.064 | 70.9  66.1  107.0  38.8  1.1  1.0 | 0.24  0.97  0.17  0.085  0.06  0.08 | 7.7  31.2  5.4  2.7  1.9  2.5 |
| B05.10  mfs3OE | -  - | | -  - | -  - | -  - | 0.062  0.098 | | 1.0  1.5 | 0.031  0.087 | 1  2.8 |

^1^ Resistance Factor (RF) values were calculated by dividing the isolate EC_50_ value by the EC_50_ value of the reference strain B05.10
