# Supplementary material for: Transcriptomic and functional analyses on a Botrytis cinerea multidrug‐resistant (MDR) strain provides new insights into the potential molecular mechanisms of MDR and fitness
Source: Mol Plant Pathol. 2024 Sep 7;25(9):e70004. doi: 10.1111/mpp.70004 (PMC11380696; doi:10.1111/mpp.70004)
Supplement: Supplementary file 9 — TABLE S3. Primers used in the current study. [file MPP-25-e70004-s008.docx]

| Table S3. List of primers used in this study | | | |
| --- | --- | --- | --- |
| Primer name | Primer sequences (5’-3’) | purpose | Fragment length (bp) |
| UCE | Fw- ATCACCCAAACATCAACT  Rv- CATAGAGCAGATGGACAA | RT-qPCR | 102 |
| mfsUP | Fw- ATGGCAGATCCCGGAATCAA  Rv- ACACCGGGCAATTCAACAAT | RT-qPCR | 118 |
| mfsOE | Fw-CATCACCCATGGCAGATCCCGGA  Rv- TCAGCCCCCTATGCGTTGTTAGAAGC | overexpression  of *Bcmfs3* | 1734 |
| pRFHUE | Fw-ACGCATAGGGGGCTGAGGACTTA  Rv-TCTGCCATGGGTGATGTCTGCTC | vector amplification | 9429 |
